# Genome-wide joint SNP and CNV analysis of aortic root diameter in African Americans: the HyperGEN study

**DOI:** 10.1186/1755-8794-4-4

**Published:** 2011-01-11

**Authors:** Nathan E Wineinger, Amit Patki, Kristin J Meyers, Ulrich Broeckel, Charles C Gu, DC Rao, Richard B Devereux, Donna K Arnett, Hemant K Tiwari

**Affiliations:** 1 Department of Biostatistics, University of Alabama at Birmingham, Birmingham, AL, USA; 2 Department of Population Health Sciences, University of Wisconsin-Madison, Madison, WI, USA; 3 Department of Pediatrics, Medical College of Wisconsin, Milwaulkee, WI, USA; 4 Division of Biostatistics, Washington University, St. Louis, MO, USA; 5 Division of Cardiology, Weill Cornell Medical College, New York, NY, USA; 6 Department of Epidemiology, University of Alabama at Birmingham, Birmingham, AL, USA

## Abstract

**Background:**

Aortic root diameter is a clinically relevant trait due to its known relationship with the pathogenesis of aortic regurgitation and risk for aortic dissection. African Americans are an understudied population despite a particularly high burden of cardiovascular diseases. We report a genome-wide association study on aortic root diameter among African Americans enrolled in the HyperGEN study. We invoked a two-stage, mixed model procedure to jointly identify SNP allele and copy number variation effects.

**Results:**

Results suggest novel genetic contributors along a large region between the *CRCP *and *KCTD7 *genes on chromosome 7 (p = 4.26 × 10^**-7**^); and the *SIRPA *and *PDYN *genes on chromosome 20 (p = 3.28 × 10^**-8**^).

**Conclusions:**

The regions we discovered are candidates for future studies on cardiovascular outcomes, particularly in African Americans. The methods we employed can also provide an outline for genetic researchers interested in jointly testing SNP and CNV effects and/or applying mixed model procedures on a genome-wide scale.

## Background

African Americans are known to be at increased risk for adverse cardiovascular outcomes including arterial stiffness and hypertension [1,2]. This observation makes African Americans a favorable population for genome-wide association studies (GWAS) on traits related to cardiovascular disease. The size of the aortic root is one such trait as it has been shown to play a major role in the pathogenesis of aortic regurgitation and risk for aortic dissection [3]. Enlargement of the aortic root has also been shown to be associated with arterial blood pressure in some studies [4-8] but not others [9] leading some investigators to hypothesize its role in the development of hypertension [10,11]. Despite reports of high heritability in common cardiovascular diseases, even large-scale GWAS have failed to replicate positive associations [12-14]. One possible explanation is that these complex diseases may be mediated by multiple pathophysiologic processes, such as enlargement of the aortic root, which are potentially better suited for disease-gene association testing. Previous studies have reported linkage between the diameter of the aortic root and candidate genes by using an extreme-values approach [15]; and in a genome scan [16]. A meta-analysis of GWAS results has identified genetic variants associated with the trait on chromosomes 5, 12p, 12q, and 17 [17]. However, only individuals of European ancestry were included.

The success of GWAS relies on the assumption that the heritability in common diseases can be captured by relatively few common genetic variants in the form of single nucleotide polymorphisms (SNPs) - sometimes referred to as the common disease, common variant hypothesis [18,19]. However, a substantial proportion of the heritability of many of these diseases remains left unexplained as traditional GWAS based upon SNPs have only accounted for a modest proportion of the total genetic variation. Among others, copy number variation (CNV) has been cited as a potential source of this so-called missing heritability [20].

Two recent studies have investigated the genomic architecture of CNVs in African Americans [21,22]. These reports suggest CNVs represent a significant source of genetic variation in this population. We report the results from a non-traditional GWAS on aortic root diameter in African Americans which evaluated SNP and copy number effects simultaneously. We gathered phenotypic and genetic data on 1,086 family members enrolled in the HyperGEN study [23] who were genotyped on the Affymetrix 6.0 array. We obtained SNP and CNV calls from the Larry Bird application in Birdsuite software [24]; and performed a two-stage, mixed model procedure designed to identify SNP and CNV effects after controlling for potential confounders. We found two interesting signals: one stretching between the *CRCP *and *KCTD7 *genes on chromosome 7 and the other between the *SIRPA *and *PDYN *genes on chromosome 20.

## Methods

Phenotypic data was obtained on 1,086 self-reported African American family members and individuals enrolled in the HyperGEN study [23]. HyperGEN is one of four Family Blood Pressure Program networks supported by the National Heart, Lung, and Blood Institute (NHLBI) to identify genetic contributors to hypertension. Subjects were recruited from centers located in Birmingham, AL and Forsyth County, NC. In the first recruitment phase of the HyperGEN study, hypertensive sibships eligible for recruitment consisted of probands with onset of hypertension by age 60 and one or more hypertensive siblings who were willing to participate in the study. In the second phase, the offspring of the hypertensive siblings were recruited. Hypertension was defined as an average systolic blood pressure ≥ 140 mm Hg or an average diastolic blood pressure ≥ 90 mm Hg during at least two evaluations, or receiving medical treatment for hypertension.

The present study cohort consisted of 421 families. Families varied in size from single individuals to larger families, up to 10 (Table 1). Measurements were recorded on age, gender, height, weight, body mass index, systolic blood pressure, diastolic blood pressure, and aortic root diameter (Table 2). Aortic root diameter measurements were performed during quiet respiration with two-dimensional echocardiography. Aortic root was evaluated at end-diastole at the level of the aortic annulus and the sinuses of Valsalva in the long-axis view [25]. Measurements were made at Cornell Medical Center and verified by an experienced investigator. Further procedures for evaluating aortic root dimensions at the sinuses of Valsalva are described in Roman *et al. *[26,27]. This study was approved by the Institutional Review Board (IRB) and all subjects provided informed consent.

**Table 1:** Family structure of African American study participants enrolled in HyperGEN and genotyped on the Affymetrix 6.0 array.

Family size	Families	Individuals
1	99	99
2	160	320
3	69	207
4	48	192
5	23	115
6	10	60
7	8	56
8	0	0
9	3	27
10	1	10

Total	421	1,086

**Table 2:** Descriptive statistics of African American study participants enrolled in HyperGEN and genotyped on the Affymetrix 6.0 array.

	n = 1,086
Age (years)	43.9 ± 13.4
Females (%)	66.6
Height (cm)	168.0 ± 90.0
Weight (kg)	91.3 ± 23.9
BMI (kg/m^**2**^)	32.5 ± 8.1
SBP (mmHg)	128.9 ± 22.2
DBP (mmHg)	73.8 ± 11.7
Center	
Birmingham, AL	856
Forsyth County, NC	230
ARD (cm)	3.26 ± 0.38

Mean ± standard deviation when applicable. BMI, body mass index; SBP, systolic blood pressure; DPB, diastolic blood pressure; ARD, aortic root diameter.

### Genotyping procedures

Genetic data was obtained through the Affymetrix Genome-Wide Human SNP Array 6.0. Samples were grouped into 34 batches in which a batch consisted of all the samples that were processed on a particular day. The Affymetrix genotyping protocol was followed. Quality control was assessed using fifty control SNPs located on each array which were typed using a second independent platform (ABI Taqman). Genotype-based quality control was assessed by checking markers for Mendelian inconsistencies, potential patterns of missing data, and allele frequency measurements (Additional file 1, S1).

SNPs and CNVs were called using Birdsuite software, Version 1.5.5 [24]. Software parameters were kept at the developers' default values; and the Human Genome 18 reference build was used for probe localization. Samples were processed by batch to eliminate batch effects. This allows for better clustering of the data and improves sensitivity and specificity of the algorithm compared to combining data across batches [28]. The Larry Bird application within Birdsuite was used to generate CNV-SNP genotypes at each locus. Larry Bird combines information on copy number segment calls using the HMM-based Birdseye, and SNP calls using the Birdseed applications within Birdsuite. The result is a more accurate depiction of locus-specific nucleotide frequency, particularly in non-diploid regions; and allows for the joint modeling of allelic and copy number effects at a given locus. Only calls with confidence values less than 0.1 were considered per the developers' recommendation (higher values indicate more uncertainty).

### Statistical analyses

SNP and CNV joint tests of genetic association on the logarithm of aortic root diameter (AR2D) were conducted on all autosomal SNP marker loci genotyped on the Affymetrix 6.0 array that passed quality control thresholds (Additional file 1, S1). The joint test was performed following an approach similar to the model proposed by Korn *et al. *[24]. That is, at any particular locus containing SNP alleles labeled generically as A and B:

(1) $$\text{Y}=\alpha +\beta_{1}\left(\text{a}-\text{b}\right)+\beta_{2}\left(\text{a}+\text{b}\right)+\varepsilon$$

where Y represents the dependent variable of interest, a is the number of A alleles at the locus, b is the number of B alleles at the locus, *β*_1 _corresponds to the additive SNP allele effect, and *β*_2 _corresponds to the additive copy number effect. The joint test involves assessing the null hypothesis: *β*_1 _= *β*_2 _= 0. When either SNP allele or copy number state is invariant, the model defaults to a single test of either allelic or copy number effect: *β*_1 _= 0 or *β*_2 _= 0, respectively.

The model described in equation {1} can be manipulated to give investigators control over potential confounding factors by including them as covariates. Because the HyperGEN cohort includes family data, the model was altered into a mixed model by including random effects for each family. Furthermore, population stratification due to genetic admixture can confound population association studies or produce spurious results [29-32]. To account for this problem, the first four principal components were included in the model as fixed effects covariates [33]. The choice of four principal components was based on examination of the associated eigenvalues (Additional file 2, Table S1). Finally, age, age-squared, sex, and the recruitment center were included as additional fixed effects covariates. Thus, the full mixed model used for the analysis can be written as:

(2) $$\begin{array}{c} \text{ln}\left(\text{AR2D}\right)=\alpha +\beta_{1}\left(\text{a}-\text{b}\right)+\beta_{2}\left(\text{a}+\text{b}\right)\\ +\sum_{j=1}^{4}\phi_{j}\left({\text{PC}}_{j}\right)+\phi_{5}\left(\text{Age}\right)\\ +\phi_{6}\left({\text{Age}}^{\text{2}}\right)+\phi_{7}\left(\text{Sex}\right)\\ +\phi_{8}\left(\text{Center}\right)+\gamma_{i}\left(\text{Family}\right)+\varepsilon \end{array}$$

where, in addition to the parameters described in {1}, *ϕ*_*j*_, j = 1,...,4 are the fixed effects from the first four principal components, *ϕ*_5_, *ϕ*_6_, *ϕ*_7_, *ϕ*_8 _and are the fixed effects for age, age-squared, sex, and recruitment center, respectively, and *γ*_*i *_is the family random effect of the *i*^th ^subject.

Fitting the mixed model described in equation {2} for each SNP marker locus is currently infeasible without approximations [34]. Because of this limitation, a two-stage procedure was enacted. In the first stage, a test of association using the GRAMMAR approach introduced by Aulchenko *et al. *[35] was used to determine the 1,000 most-likely significant markers. In the second stage, those 1,000 markers, referred to as the top 1,000, were tested using the full mixed model described in equation {2}. The GRAMMAR approach allows genome-wide mixed model calculations to be computed on a reasonable timescale. As part of the procedure, model residuals for aortic root diameter measurements are generated via mixed model after controlling for relatedness and fixed effect covariates. However, genetic effects are not included. In terms of the present study:

(3) $$\begin{array}{c} \hat{\varepsilon_{i}}=\ln\left({\text{AR2D}}_{i}\right)-\hat{\alpha }-\sum_{j=1}^{4}\hat{\phi_{j}}\cdot {\text{PC}}_{i,j}\\ -\hat{\phi_{5}}\cdot {\text{age}}_{i}-\hat{\phi_{6}}\cdot {\text{age}}_{i}^{2}-\hat{\phi_{7}}\cdot {\text{sex}}_{\text{i}}\\ -\hat{\phi_{8}}\cdot {\text{center}}_{i}-\hat{\gamma_{i}}\cdot {\text{family}}_{i}\\ ={\text{Y}}_{i}^{*} \end{array}$$

The resulting residuals (${\text{Y}}_{i}^{*}$) for the *i*^th ^subject are then regressed against his or her SNP allele and copy number state at each locus in a linear model:

(4) $${\text{Y}}^{\text{*}}=\alpha +\beta_{1}\left(\text{a}-\text{b}\right)+\beta_{2}\left(\text{a}+\text{b}\right)+\varepsilon .$$

**Figure 1:**
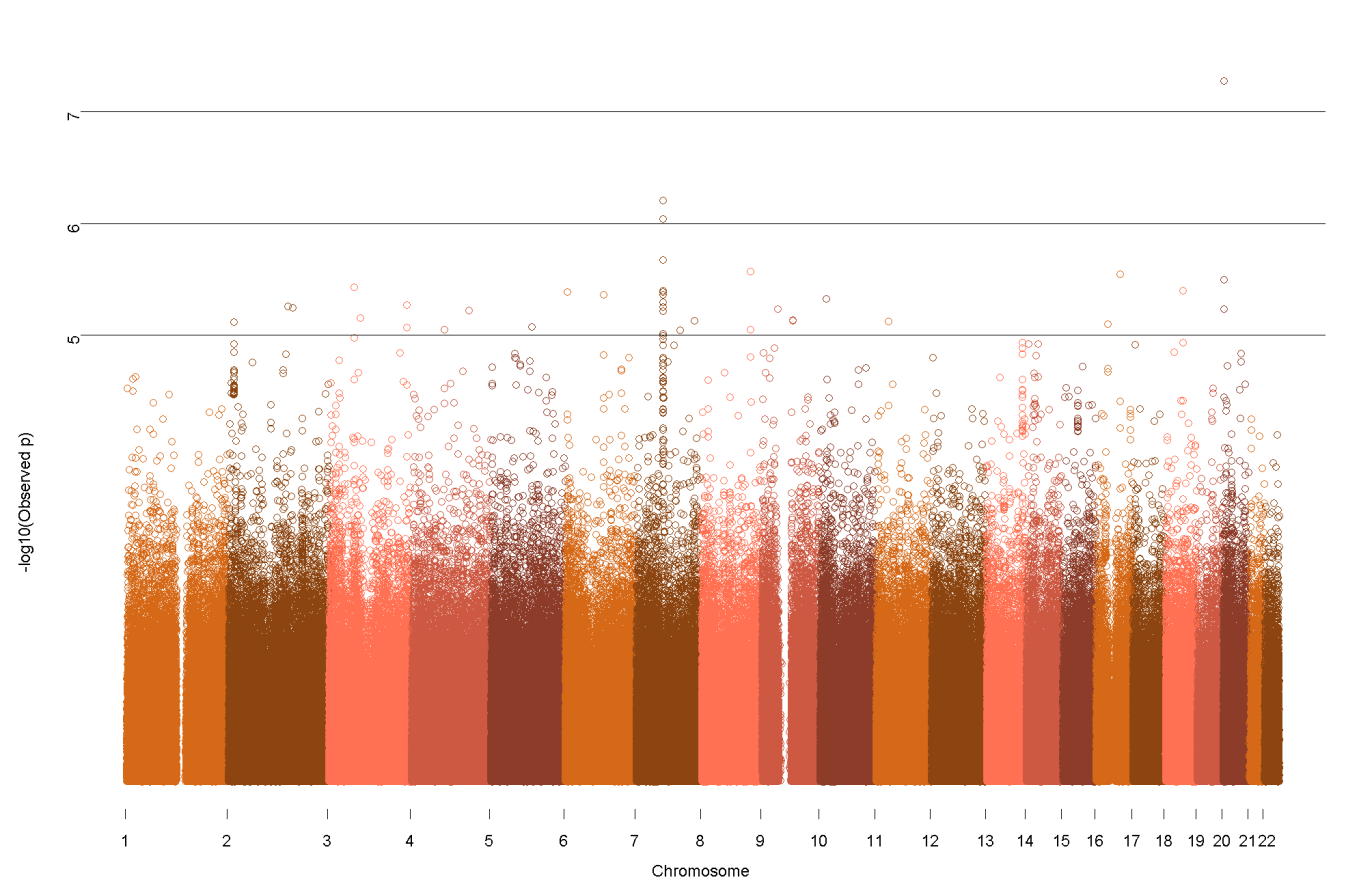
**Genome-wide results from the GRAMMAR procedure (first stage)**. Chromosomes are separated by color.

Residuals for use in the GRAMMAR approach were obtained from the PROC MIXED procedure in SAS^® ^software, Version 9.1.2 (SAS Institute Inc., Cary, NC). The residuals were regressed on allelic and copy number state via *lm *using R version 2.10.1 [36]. Genome-wide measurements were recorded (Figure 1). The markers corresponding to the 1,000 most-likely significant findings using this approach were recorded; and only these were analyzed in the full mixed model described in equation {2}, using the *lmer *function in R [37]. The most promising results are included in Table 3 and the second stage results from all loci in the top 1,000 are included in Additional file 3, Table S2. Genetic markers with p-values less than 5 × 10^-7 ^were defined as genome-wide significant [12,17].

**Table 3:** The top ten most significant markers from the first and second stages of the aortic root diameter genome-wide association study.

				Stage 1	Stage 2		
Chromosome	Base pair	Marker	MAF	p-value	SNP effect*	p-value	Gene(s)
3	59,877,841	rs1825630	0.444	3.70 × 10^-5^	9.46 (2.11)	7.99 × 10^-6^	*FHIT*
7	65,255,030	rs875971	0.324	3.99 × 10^-5^	10.69 (2.24)	4.09 × 10^-6^	*CRCP*
7	65,668,047	rs801193	0.337	3.97 × 10^-5^	11.13 (2.23)	3.65 × 10^-6^	LOC401365, LOC493754
7	65,700,370	rs10258739	0.318	2.11 × 10^-5^	10.64 (2.26)	2.50 × 10^-6^	
7	65,733,463	AFFX 9317457	0.332	6.24 × 10^-6^	11.25 (2.23)	6.20 × 10^-7^	*KCTD7*, *RABGEF1*
7	65,733,463	rs10263935	0.324	9.06 × 10^-6^	11.42 (2.28)	4.26 × 10^-7^	*KCTD7, RABGEF1*
7	65,790,536	rs2659915	0.334	4.10 × 10^-5^	10.84 (2.22)	4.27 × 10^-6^	*RABGEF1*
8	117,625,451	rs4876662	0.195	2.70 × 10^-5^	13.17 (2.58)	2.23 × 10^-6^	
16	57,316,833	rs12600277	0.162	2.84 × 10^-5^	13.99 (2.98)	2.72 × 10^-6^	*GOT2*
20	1,888,504	rs6045666	0.351	3.21 × 10^-5^	12.58 (2.26)	2.97 × 10^-6^	*SIRPA, PDYN*†
20	1,889,171	rs6045676	0.356	5.31 × 10^-7^	11.47 (2.28)	3.28 × 10^-8^	*SIRPA, PDYN*†

The markers at rs6045676 and rs10263935 reach the threshold for genome-wide significance (p < 5 × 10^-7^). The last column lists known genes and proteins of unknown function within 10 kb of the identified marker.

* The regression coefficients (10^-2 ^scale) for the SNP effect in equation {2} and its standard error (in parentheses) are included. Meanwhile, CNV effects are excluded due to the low observed CNV frequency at each marker (<1%).

† These genes are outside the 10 kb restriction, yet still included.

## Results and Discussion

Genome-wide results were generated via a two-stage procedure (see Methods). The GRAMMAR procedure (first stage), alone, does not provide accurate parameter estimates and significance values. The systematic depression of the test statistic, which can be seen in the quantile-quantile (QQ) plot (Additional file 4, Figure S1), demonstrates that this method is inherently conservative. This finding agreed with published results [35]. Due to this limitation we implemented the GRAMMAR approach as a screening procedure. In this first stage, the top 1,000 loci were identified. These loci included all markers with p-values less than 2.5 × 10^-3^. In general, these markers appeared to be uniform across the genome, with the exception of a few regions. Chromosome 7, in particular, had numerous markers making it into the top 1,000. Many of these markers were found in a 500 kb region spanning the *CRCP *and *KCTD7 *genes. It should be noted that neither loci within genes previously found to be associated with thoracic aortic aneurysm (*ACTA2, MYH11, TGFBR1*, and *TGFBR2*), nor regions previously identified as being associated with aortic root size reached inclusion into the top 1,000 [17].

**Figure 2:**
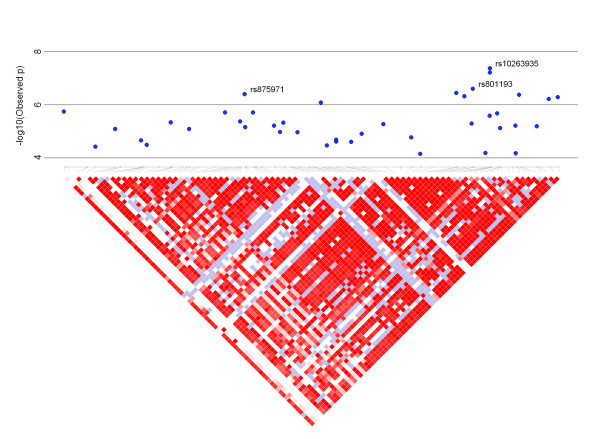
**Inspection of significance peak on chromosome 7**. Linkage disequilibrium measurements (below) overlaid with results from the full mixed model (second stage) on chromosome 7: 64,902,615 - 65,866,167 (above).

As the results from the GRAMMAR procedure, alone, are not conclusive, we re-analyzed the top 1,000 from the first stage using the full mixed model described in equation {2}. We identified two regions of significance: the strongest signal found between the *SIRPA *and *PDYN *genes on chromosome 20; and numerous signals within the 500 kb region on chromosome 7 highlighted in the first stage results. In total there were 24 marker loci with p-values less than 1 × 10^-5^. Many of these were located within the 500 kb region on chromosome 7 (Figure 2), but a few were found elsewhere (Table 3).

While the loci that reached or approached genome-wide significance predominantly achieved that threshold based upon SNP effects, the second largest signal, found within the *KCTD7 *gene on chromosome 7, was influenced, in part, by a rare copy number variant (less than 1%). The copy number estimate at this locus was nearly three times its standard error. Also, a 40 kb copy number variable region on chromosome 2 within a gene gap between *LPIN1 *and *TRIB2 *genes showed relatively large copy number effect estimates - about four times its standard error. At this location, a rare deletion was correlated with higher aortic root diameter (p_CNV _= 2.97 × 10^-5^).

## Conclusion

African Americans have been disproportionately represented in genetic association studies on cardiovascular traits despite a well-documented high burden of cardiovascular diseases. We implemented a non-traditional statistical approach to test for genetic associations with aortic root diameter in African American family members enrolled in the HyperGEN study. This method allowed us to jointly test for SNP and CNV effect while controlling for family structure.

We identified two novel regions of genetic association on chromosomes 7 and 20. The significant SNPs in each region (rs10263935 and rs6045676, respectively) have different allele frequencies in YRI and CEU populations in HapMap [38] - providing a possible explanation why these associations have not been observed in previous studies based on populations of European descent [17]. The single strongest signal of genetic association was found on chromosome 20 between the *SIRPA *and *PDYN *genes. However, a clear relationship with either gene to cardiovascular traits beyond the results of this study has not been discovered. *SIRPA *is involved in negative regulation with numerous growth factor signaling receptors; and *PDYN *has been found to be associated with nonlesional temporal lobe epilepsy [39], but results have not been replicated [40]. Meanwhile, the much wider region identified on chromosome 7 contains an interesting gene that has been previously found to be related to cardiovascular outcomes: *CRCP*. The product of the *CRCP *gene is known to interact with *CRLR *to facilitate adrenomedullin (ADM) mediated signaling [41]. *CRLR *has been found to be significantly decreased in the umbilical artery and uterus of women with pregnancy-induced hypertension [42]. Also, post-translationally modified ADM has been known to influence vasodilation [43].

The two-stage procedure, joint analysis, and ascertainment scheme offered some limitations to the present study. First, it is possible that we missed a true association that did not pass the first stage criteria; but after comparing the results between the loci that were included in both steps, we are fairly comfortable as the results were consistent across stages. Only three of the top 1,000 did not have p-values less than 3 × 10^-3 ^in the second stage; and among the most significant findings, nine were in the top ten most significant findings in both stages. Second, the joint analyses we performed frequently reverted back to a conventional SNP analysis due to the majority of the genome being copy number invariant. However, we were cautious about using a SNP-only approach throughout the genome as it would require us to either treat non-two-copy genotypes as missing, or force them into a two-copy state. And finally, the HyperGEN sib-pair ascertainment scheme restricted our ability to distinguish inherited copy number variation from variants arisen through *de novo *mutation as the direct transmission of CNVs from both parents to offspring was unobservable.

We found no findings that proved copy number variation was playing a role in the pathogenesis of aortic root diameter, but we do not believe CNVs should be ruled out altogether. In a few cases we found large estimates with promising significance values. Unfortunately, the low frequency of copy number events restricted our ability to separate their effects from more frequent SNPs. Also, copy number variants potentially cover numerous loci. In terms of copy number alone, we expect perfect or near-perfect linkage disequilibrium among those loci. Therefore, perhaps the same criteria imposed for genome-wide significance on SNPs should not hold for CNVs when looking at a "per locus" level.

Among those genes we identified as potentially influencing aortic root diameter, *CRCP *perhaps shows the most promise. However, the large region encompassing this gene shows strong patterns of linkage disequilibrium (Figure 2). Numerous other neighboring genes or loci could be influencing the result. Our other primary finding on chromosome 20 is curious as the flanking genes and reported expression results have not previously identified this region as contributing to cardiovascular outcomes. Nonetheless, future genetic studies on aortic root diameter and other related cardiovascular traits should consider the regions we identified as candidates.

## Competing interests

The authors declare that they have no competing interests.

## Authors' contributions

NEW obtained genotype calls from the processed data, performed the tests of association in both stages of the analysis, and prepared the manuscript. AP obtained principal components and was involved in database management. KJM performed tests of association on chromosome 7 on an independent dataset. UB converted raw data to processed genotypes. CCG, DCR, RBD, and DKA are involved with the recruitment and upkeep of the HyperGEN cohort. HKT assisted in preparation of the manuscript. All authors read and approved the final manuscript.

## Pre-publication history

The pre-publication history for this paper can be accessed here:

http://www.biomedcentral.com/1755-8794/4/4/prepub

## Supplementary Material

Additional file 1 **S1**. SNP quality control and principal component analysis. Click here for file

Additional file 2 **Table S1**. Eigenvalues of the top 30 principal components. Click here for file

Additional file 3 **Table S2**. Top 1,000 results from the second stage of the analysis. Click here for file

Additional file 4 **Figure S1**. Quantile-quantile plot of genome-wide results from the first stage (GRAMMAR) procedure. Click here for file
