# Supplementary material for: Genome-wide joint SNP and CNV analysis of aortic root diameter in African Americans: the HyperGEN study
Source: BMC Med Genomics. 2011 Jan 11;4:4. doi: 10.1186/1755-8794-4-4 (PMC3027088; doi:10.1186/1755-8794-4-4)
Supplement: Additional file 1 — S1. SNP quality control and principal component analysis. [file 1755-8794-4-4-S1.PDF]

S1

SNP marker quality control thresholds

Only SNP markers that passed quality control thresholds were included in both stages of the analysis. All markers that failed at least one quality control threshold were removed from consideration in the second stage of the analysis. SNPs were removed based on Mendelian inheritance errors (overall error rate = 0.045%), excessive missingness (threshold: missingness > 1%; n = 282), low minor allele frequency in founders (threshold: minor allele frequency less than 1%; n = 14,541), and failure of Hardy-Weinberg equilibrium in founders (threshold: $p < 0.001$; n = 7,879).

Choice of principal components

We performed principal component analysis (PCA) to control for potential confounding due to admixture. We considered up to thirty principal components (PC) as covariates in the model described in equation {2}. While the first PC is often interpreted as the degree of African ancestry, we felt that including only this term may be insufficient due to what we knew about the study population (different geographic regions) and ascertainment scheme, as well as our uncertainty about the ancestral origins in the sample. Therefore, rather than *a priori* choosing an arbitrary number of PCs to include, we chose to let the data guide us to a decision.

We arrived at the choice of the first four PCs because the eigenvalue associated with the fourth PC fell into what we believed to be the "elbow" of the scree plot (Supplemental Table 1). That is, the relative decrease in eigenvalues from one PC to the next was considerably and consistently smaller beyond the fourth PC.

By including PCs beyond the first, it is possible that we are over-adjusting the model as we are also incorporating family random effects and recruitment center effects – both which

likely include a polygenetic component. However, the correlation between these PCs and family, and center was low (each less than 0.025 and 0.004, respectively).

We examined the residuals obtained from equation {3} and compared them with the residuals we would have obtained had we chosen to include various numbers of PCs as covariates. For all cases, the correlation was high ($r^2 > 0.99$), indicating the results would have been very similar had we chosen a different number of PCs. At the conclusion of our study we also re-examined our top hits in Table 3 after considering different numbers of PCs. The results we obtained were consistent with our original discoveries.
