# Supplementary material for: Genome-wide joint SNP and CNV analysis of aortic root diameter in African Americans: the HyperGEN study
Source: BMC Med Genomics. 2011 Jan 11;4:4. doi: 10.1186/1755-8794-4-4 (PMC3027088; doi:10.1186/1755-8794-4-4)
Supplement: Additional file 4 — Figure S1. Quantile-quantile plot of genome-wide results from the first stage (GRAMMAR) procedure. [file 1755-8794-4-4-S4.DOCX]

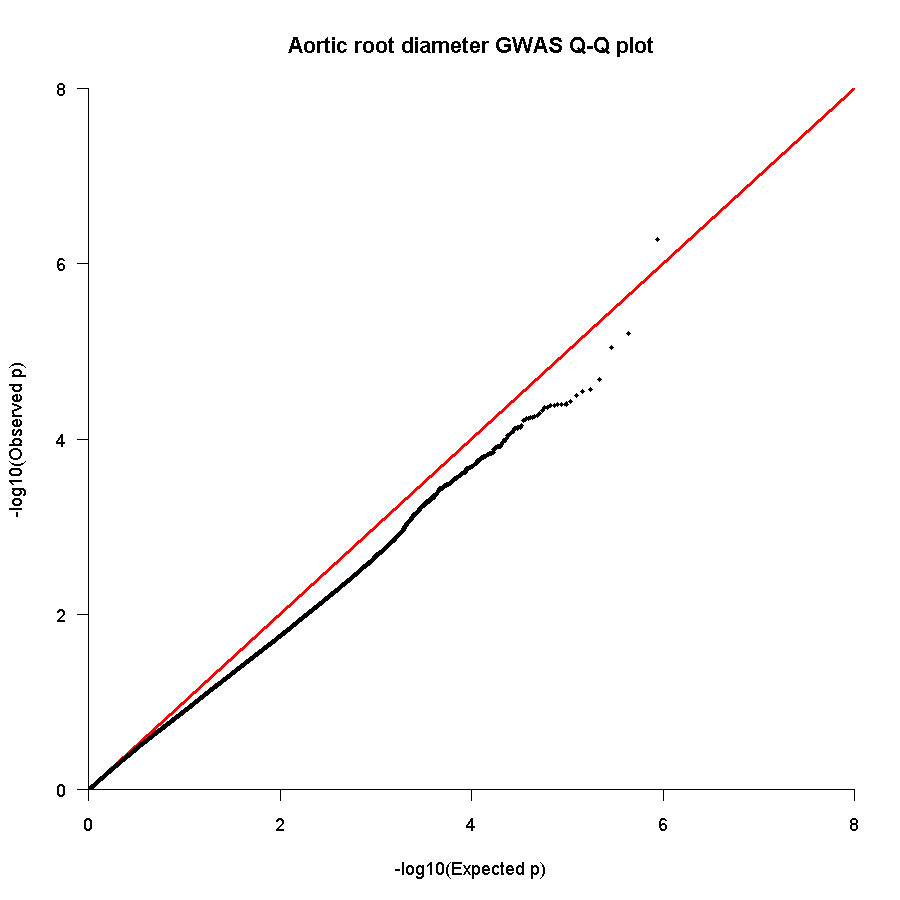


**Figure S1 - Quantile-quantile plot of genome-wide results from the GRAMMAR procedure (first stage).** The discrepancy between the observed and expected p-values is due to the conservative nature of this approach.
